# Relation classification via BERT with piecewise convolution and focal loss

**DOI:** 10.1371/journal.pone.0257092

**Published:** 2021-09-10

**Authors:** Jianyi Liu, Xi Duan, Ru Zhang, Youqiang Sun, Lei Guan, Bingjie Lin

**Affiliations:** 1 School of Cyberspace Security, Beijing University of Posts and Telecommunications, Beijing, China; 2 School of Electrical Engineering, Tsinghua University, Beijing, China; 3 State Grid Information and Telecommunication Branch, Beijing, China; Taipei Medical University, TAIWAN

## Abstract

Recent relation extraction models’ architecture are evolved from the shallow neural networks to natural language model, such as convolutional neural networks or recurrent neural networks to Bert. However, these methods did not consider the semantic information in the sequence or the distance dependence problem, the internal semantic information may contain the useful knowledge which can help relation classification. Focus on these problems, this paper proposed a BERT-based relation classification method. Compare with the existing Bert-based architecture, the proposed model can obtain the internal semantic information between entity pair and solve the distance semantic dependence better. The pre-trained BERT model after fine tuning is used in this paper to abstract the semantic representation of sequence, then adopt the piecewise convolution to obtain semantic information which influence the extraction results. Compare with the existing methods, the proposed method can achieve a better accuracy on relational extraction task because of the internal semantic information extracted in the sequence. While, the generalization ability is still a problem that cannot be ignored, and the numbers of the relationships are difference between different categories. In this paper, the focal loss function is adopted to solve this problem by assigning a heavy weight to less number or hard classify categories. Finally, comparing with the existing methods, the F1 metric of the proposed method can reach a superior result 89.95% on the SemEval-2010 Task 8 dataset.

## Introduction

With the advent of the intelligent era, information extraction technology is gradually used to process the massive text data on the Internet and digital platform. This technology can get the information which is needed and filtering a lot of spams. The information extraction is designed to extract structured information from large-scale unstructured or semi structured natural language texts. Relationship extraction is one of the important sub tasks, which aims to identify entities from text and extract semantic relations between entities. As one of the sub tasks of information extraction, relation extraction (RE) is widely concerned by academia and industry and it is widely used in information retrieval [1], automatic question and answer [2], knowledge base construction [3], semantic network, knowledge graph [4], visual question answering [5, 6] and other fields. RE extracts the semantic relation between entity pairs from the sequence, such as the relationship between whole and part (part-whole), belonging relationship (belongs-to), employment relationship (employee-of) and so on, and combine the extracted relationship and entities into triples <*Entity*_1_, *Relation*, *Entity*_2_> to prepare for other tasks.

With the application of deep learning (DL) in various fields, the task of relation extraction is changing from pattern matching and machine learning to deep learning. Compare with the traditional methods, the deep learning can automatic extract features and achieve a better performance. So, the deep learning methods are gradually replacing traditional methods. However, in the processing of relation extraction, the existing deep learning methods are still not rational, which cannot extract essential semantic features. In addition, the relationship extraction is usually adapted in a specific domain, which will cause the extraction model’s vulnerability in generalization. And the scalability and portability are not satisfied, it cannot be transplanted or expanded in different fields according to needs. In the supervised relation extraction task, the input features are needed design by researchers, such as POS (part-of-speech), dependency parsing and so on. And the existing extraction model which can’t understand the essential semantic relationship of sequences, and most methods are not considering the data imbalance problem. So, in this paper, we focus on the extract precision and data balance, and propose an extraction method based on pre-trained Bert with piecewise convolution and focal loss.

Recently, the extraction methods gradually adopt deep learning than traditional methods. The CNN based relation classification is first proposed in 2014 [7], and the attention mechanism Attention and recurrent neural network Bi-LSTM are first used in 2017 to jointly extract entity and classification relations. In addition, remotely supervised entity relation extraction methods based on deep learning mainly include network structures such as CNN, RNN, and LSTM [8–10].

In recent years, scholars have proposed a variety of improvements on top of the basic methods, such as the fusion method of PCNN and multi-instance learning [11], the fusion method of PCNN and attention mechanism [12], etc. A piecewise convolutional neural network (Piecewise Convolutional Neural Networks, PCNNs) model based on multi-instance learning (MIL) is proposed for relation extraction tasks [11]. The convolutional neural network models (APCNNs) based on the attention mechanism is proposed for the problem of data noise by [13]. The COTYPE model proposed by [14] and the residual network proposed by [15] both enhance the relation extraction effect. These methods are based on supervised learning, which can solve the problem of wrong label and error propagation in traditional methods. However, these universal DL-based methods have the problems such as difficulty in selection of initial seed and candidate relation pattern, and exist semantic drift.

The existing models only used the feature information of the entity pair, the semantic information which between the entities are ignored. While, the ignored information is useful to relation classification, and contains a lot of information between relations. The BERT model which has proven to be very effective for improving many natural language processing tasks [16], and focus on the feature extraction of remaining segments, the piecewise convolution has better performance in multi-classification task. So, to solve the problems in existing methods, we proposed a relation extraction method which used the Bert and piecewise convolution. The innovations of this paper are as follows:

1. Compare with the CNN and RNN model, the Bert model have achieved a superior result in NLP. But the existing Bert-based relation extraction models have not used the semantic information between the entities. To extract the semantic information between the entities, the sequence is cut into three segments. And the Bert model is used to extract the features of semantic representation in the sequence, which considers the feature information of the target entity pair and the semantic information of the remaining words in the sequence;
2. The piecewise convolution performances superior in multi-label classification. The outputted vectors by Bert is a matrix, when extracting the relation features of the sequence, the representation matrix of the sequence is taken as the input of the neural network. The rows of the matrix represent the coding representation of a word, and the columns mean the contents of a sequence. Then uses the piecewise convolution to capture the features among the multiple consecutive words;
3. In order to enhance the generalization ability of the proposed model, the Focal Loss function [17] was adopted in the training process, and higher weights were assigned to the relationship classes with fewer samples or difficult to train to ensure the extracting effect.

The rest of this paper is organized as follows. Section 2 gives related work on the methods of relation extraction. The proposed model is introduced in detail in section 3. The experiments of relation extraction are given in section 4. Finally, conclusion and future work are given in section 5.

## Related work

Supervised entity relation extraction methods can be divided into the following three categories:

### The method of pattern matching

The relation extraction system (FASTUS) based on pattern matching is built by [18], and the form of matching rules is not limited to specific fields. When researchers need to apply to a specific field, they only need to set some parameters in the matching rules. The relation extraction system (Proteus) on rule-based matching is proposed by [19], and processed relation categories through human-computer interaction. The method of pattern matching is time-consuming, labor-intensive and costly because the pattern rules need to be artificially defined and summarized. At the same time, most of the rules are only suitable for a single corpus field, and the commonality between different fields is poor, and it is difficult for the system to achieve the required recall rate. The parts of the pattern-based matching models are list in Table 1.

**Table 1:** The different models and datasets based on pattern-based matching.

Extraction method	Model	Data Set	Year	Published
Pattern-based matching	FASTUS extraction system	MUC-6	1995	MUC
	Proteus extraction system	MUC-7	1998	MUC
	Semi-supervised extraction system	World Wide Web	1998	MUC

### The method based on machine learning

There are two main methods based on machine learning, according to the difference in sample processing.

The feature vector method transforms natural language units into feature vectors through a vector space model, uses the feature vectors as the input of the classifier, and provides a probability basis for classification after a series of related calculations. The Maximum Entropy Models (MaxEnt) model which combined with lexical and syntactic analysis features is proposed by [20]. But this model only processes the explicit relationships, because the agreements between annotators are poor in implicit relationships. The reference [21] combined the Naive Bayes (NB) and Multi-layer Perceptron (MLP), and proposed a feature-based method for extracting Chinese term relations. The results show that the performance of the proposed hybrid algorithm is almost better than that of the single NB algorithm and MLP algorithm. A method based on probability model and domain knowledge is proposed by [22], this paper uses encyclopedia to build a domain knowledge base, then develop a sentence simplification model to simplify the complex sentences and take the relationship extraction problem as a sequence labeling task. The limitations of these methods are that the researcher’s personal experience and background knowledge will affect the relation features selection, and these methods do not catch the semantic information of the sequence enough.

The second methods are based on the kernel function. The smallest common subtree on the syntactic tree is taken as the feature of relation extraction, and at the same time trained a machine learning classifier to classify the relation category [23]. The method [24] used the dependency syntax tree function and extended the tree kernels, then the paper used WordNet to extend the subtree matching function. The experimental results showed that using dependency tree kernel for relation extraction provides an obvious improvement over word bag kernel. A sequence kernel function based on word sequence combination is constructed by [25], then the k-Nearest Neighbor (KNN) model is used in machine learning as a classifier to classify entity relation categories. Compared with the feature-based methods, the kernel-based methods have the advantage of using long-distance features and structural features to more fully integrate semantic information. However, the learning and training speed based on the kernel function methods are slow, because of the strict matching constraints in the similarity calculation process. Table 2 shows the models based on machine learning.

**Table 2:** The different models and datasets based on machine learning.

Extraction method		Model	Dataset	Year	Published
Machine learning	Feature vector	Maximum entropy model	ACE2004	2004	ACL
		SVM (phrase information, WordNet)	ACE2004	2004	ACL
		Combining NB and MLP	Annotation data	2009	ICSPS
	Kernel function	FDRM	Annotation data	2012	PACLIC
		SVM with shallow syntactic analysis tree	NAPT	2003	MLR
		SVM with dependency syntax tree	NIST	2004	ACL
		KNN with semantic sequence kernel	ACE2005	2007	CRD

### The method based on deep learning

According to the different process of relation extraction, the semantic relation extraction method based on deep learning is divided into pipeline method and joint learning method.

The pipeline method is named according to the process of relation extraction, which recognizes the entities and then completes the relation extraction task. In the previous pipeline method, CNN and RNN are mainly used to extract the relation. CNN is advantageous to recognize the structural features of the target, and RNN is helpful to recognize the sequence features. The CNN-based model also has the advantages of fast training and parallel computing. The method [7] applies the CNN model to the task of relation extraction for the first time. The multi-layer attention mechanism of the CNN model is used in relation extraction [26]. The reference [27] obtained the long lexical information, and proposed a relation extraction method based on RNN, which assigns a vector and a matrix to each node in the analysis tree. And extract the relation in cause-effect or topic-message between nouns using the syntactic path between them. An RNN model based on a syntactic tree and allowed an explicit weighting of important phrases is proposed for the target task [28], which combines a variety of feature information, such as part-of-speech tags, syntactic headers and phrase categories. A new end-to-end recursive neural model (Entity Attention Bi-LSTM) is proposed by [29], which combines entity-aware attention mechanism and latent entity type (LET) method. The proposed model can utilize entities and their latent types as features to classify the relation. The SDP-LSTM [30] proposed a novel neural network to classify the relation of two entities in a sentence. This method used the shortest dependency path (SDP) between two entities, and verified the proposed model in SemEval 2010 dataset, 83.7% accuracy had achieved. A bidirectional long-short memory network (Bi-LSTM) model is proposed by [31], and the proposed model extracted the relation through the whole sentence. And achieved a comparable performance. The method [32] proposed a bidirectional recursive convolutional neural network (BRCNN) model combining CNN and bidirectional LSTM.

A new relation extraction method [33] is proposed based on transformer. This method uses the Transformer [34] model proposed by Google to learn the representation of the input text. The slight difference is that the original FNN layer is replaced with a CNN with a window size of 5. This method has achieved advanced results on the biomedical drug disease dataset (Chemical Disease Relations, CDR). The relation classification method [16] is proposed by using a pre-trained BERT language model [35] and target entity information. This method uses a pre-trained BERT model to obtain the coding representation corresponding to each word in the sentence, then obtains the corresponding coding representation according to the location index of the target entity, and predicts the relation category according to the target entity information. Based on transformer, the reference [36] studied the general representation of multiple relations, and the authors provide a method to learn the representation in remote supervised or unsupervised corpus. And used the BERT model to represent text relations, and proposed the Matching-the-Blanks method to pre-train the task agnostic relation extraction method. This method achieves the effect of SOTA (state of the art) on the datasets of SemEval 2010 Task8. The transformer-based pipeline methods have achieved a SOTA result, these results illustrate that the Bert model has better processing ability in sequence text. However, these methods are using the Bert model to extract the relation in the sentence, but they only use the entities’ information and ignore the sematic information between the entities. The ignored information may be the most crucial features for relation classification, and these methods are also not considering the Generalization ability.

The joint learning method uses the model to directly extract entities and relations. A two-way LSTM model [37] which is the first true neural network joint extraction method, and this model added an attention mechanism for joint extraction. The model [38] first applied the neural network to the end-to-end entity and relation joint extraction. The proposed model can detect the entities during the training, and use the entities when extract the relation from sentences. A joint extraction method based on a deep bidirectional LSTM model [39] is proposed, it can improve the extraction performance when adding the sentence-level and relation-level to output layer. The new tagging strategy method to semantic relation extraction is applied in method [40], and convert the joint extraction task to a tagging problem. The authors researched the different end to end model to extract entities and their relations directly. These joint methods extract the entities and relations directly from the sentence, however, during the extraction processing, the association between corresponding entities still needs to be further refined. The Table 3 gives the summary of the deep-learning based models.

**Table 3:** The different models and datasets based on deep learning.

Extraction method		Model	Dataset	Year	Published
Deep learning	Pipeline	MV-RNN	SemEval-2010 Task8	2012	EMNLP
		RNN with syntactic analysis tree	Semeval-2010 Task8	2013	EMNLP
		SDT-LSTM	SemEval-2010 Task8	2015	EMNLP
		Bi-LSTM-RNN	SemEval-2010Task8	2016	ACL
		BRCNN	SemEval-2010 Task8	2016	ACL
		Entity Attention Bi-LSTM	Semeval-2010 Task8	2019	MDPI
		Convolutional DNN	SemEval-2010 Task8	2014	COLING
		CR-CNN	SemEval-2010 Task8	2015	ACL
		Multi-Attention CNN	SemEval-2010Task8	2016	ACL
		Self-attention model	CDR	2018	NAACL
		R-BERT	SemEval-2010 Task8	2019	—
		Matching-the-Blanks	SemEval-2010 Task8	2019	ACL
	Joint	Bi-LSTM + Bi-Tree LSTM	ACE2005	2016	ACL
		Deep-LSTM	MPQA 2.0	2016	ACL
		Bi-LSTM + Attention	ACE2005	2017	ACL
		Novel tagging scheme	NYT	2017	ACL

At present, the transformer-based model has achieved superior results than others, and focus on the problems which existing in the references, we proposed a relation extraction method based on Bert. Meanwhile, to ensure the extraction performance, we used the piecewise convolution to extract the features of relation, and added the focal loss during the training process. The Bert is used to vector the entities’ features and semantic information from sentences, then uses the piecewise convolution to classify the relationship between the entity pair. And to solve the data imbalance problem during the training process, the focal loss is adopted in the proposed model. To keep the relation extraction performance, the higher weight of the less sample kind and hard-training kind is set.

## Methodology

### The model architecture

**Fig 1:**
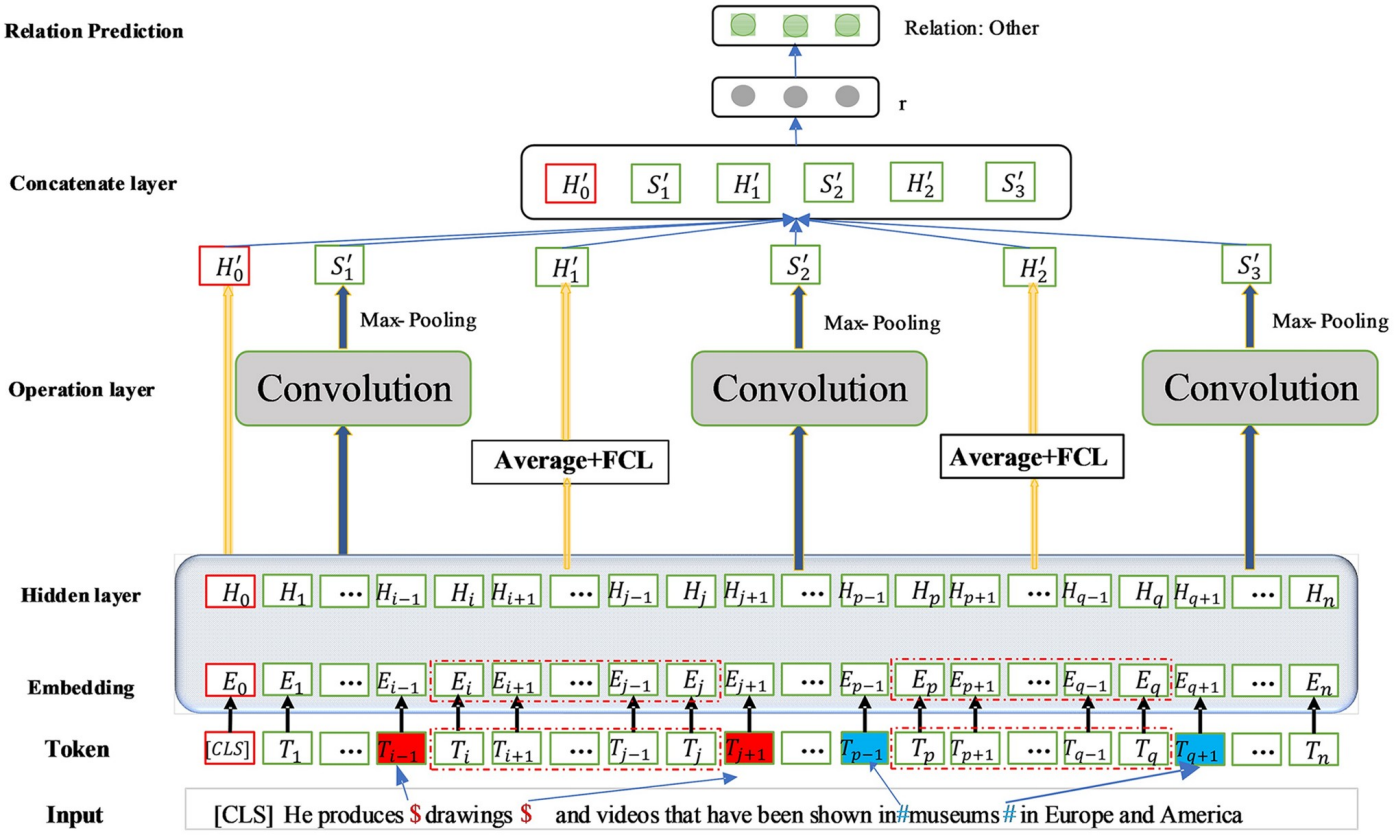
The overall model framework.

For a given sequence and two entities [*Entity*_1_, *Entity*_2_], our model will predict the probability of each relation R, and then obtain the corresponding relation category based on the probability. The model architecture is shown in Fig 1.

For a given sequence, this paper inserts the identifier $ at the beginning and end of the entity at the same time, and inserts the identifier # at the beginning and the end of the entity at the same time. This operation helps to capture the entity position. According to the input representation of the BERT model, [*CLS*] is added to the beginning of the sequence and [*SEP*] is added to the end of the sequence. Assuming that the vector is the output of the hidden layer corresponding to the head identifier [*CLS*], the vector is obtained after a fully connected layer, where the tangent function (Tanh) is used as the activation function, and the bias term is added. The calculation process is as follows Eq (1):

$$\begin{array}{c} H_{0}^{*}=W_{0}\left(\tanh\left(H_{0}\right)\right)+b{}_{0} \end{array}$$ (1)

Denote the vector sequence encoded by the entity *Entity*_1_ through the BERT model as *H*_*i*_ to *H*_*j*_, that is, the vector [*H*_*i*_, *H*_*i*+1_, …, *X*_*j*_]. The vector sequence of *Entity*_2_ encoded by the BERT model is denoted as *H*_*p*_ to *H*_*q*_, that is, the vector [*H*_*p*_, *H*_*p*+1_, …, *X*_*q*_]. The target entity pair uses the average pooling operation to obtain the final vector representation of each target entity. After the final vector representation passes through the fully connected layer, the entity *Entity*_1_ obtains the corresponding output vector representation $H_{1}^{*}$, and the entity *Entity*_2_ obtains the corresponding output vector representation $H_{2}^{*}$, both of which use the tangent function (Tanh) as the activation function and add the bias term. The calculation process is as follows Eqs (2) and (3):

$$\begin{array}{c} H_{1}^{*}=W_{1}\left(\tanh\left(\frac{1}{j-i+1}\sum_{t=i}^{j}H_{t}\right)\right)+b{}_{1} \end{array}$$ (2)

$$\begin{array}{c} H_{2}^{*}=W_{2}\left(\tanh\left(\frac{1}{q-p+1}\sum_{t=p}^{q}H_{t}\right)\right)+b{}_{2} \end{array}$$ (3)

Among them, *W*_0_ ∈ *R*^*d*×*d*^, *W*_1_ ∈ *R*^*d*×*d*^ and *W*_2_ ∈ *R*^*d*×*d*^ represent the vector dimension of the output layer of the BERT model.

### The piecewise convolution

**Fig 2:**
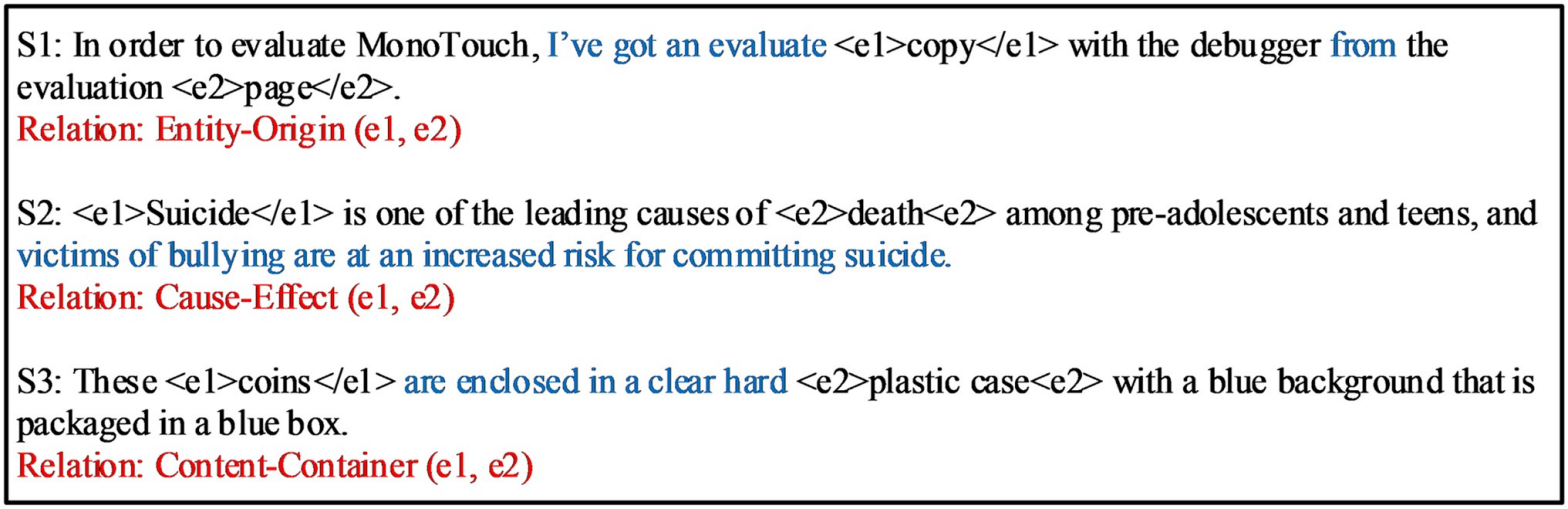
The semantic information in the sequence. This example come from SemEval2010 Task8.

Certain words or phrases in the sequence will provide necessary semantic information to illustrate the semantic relation between target entities. These words and phrases have rich expression forms and cannot be obtained by means of template matching or rule matching. For each sentence, in addition to the feature information of the entity, the words or phrases that have an impact on the classification results may be verbs, prepositions, pronouns, or verb phrases and prepositional phrases. As shown in Fig 2, through the phrase “…got…from…” in sentence *S*1 in blue font, it can be judged that the relation between the entities “copy” and “page” is the source relation, namely Entity-Origin (*e*1, *e*2). In the same way, the phrase “victims of bullying are at an increased risk for committing suicide” in sentence *S*2 in blue font explains the entity pair, and it can also be judged that the relation between the entity “Suicide” and “death” is causal, that is Cause-Effect (*e*1, *e*2). Similarly, the phrase “…are enclosed in…” in blue font in sentence *S*3 can determine that the relation between the entities “coins” and “plastic case” is the containment relation, that is, Content-Container (*e*1, *e*2).

Therefore, when doing relation classification, not only the feature information of the target entity pair needs to be considered, but also the semantic information contained in the words or phrases in the sequence.

Referring to the convolutional neural network architecture of references [41, 42], let $x_{i}\in R^{k}$ denote the k-dimensional encoding representation of the *ith* word in the sentence. A sequence of length n (fill if necessary) means:

$$\begin{array}{c} X_{i:n}=X_{1}⊕X_{2}⊕X_{3}⊕\ldots ⊕X_{n} \end{array}$$ (4)

Where ⊕ represents the concatenation operator, usually let *X*_*i*:*i*+*j*_ represent the concatenation of sequence *X*_*i*_, *X*_*i*+1_, …, *X*_*i*+*j*_. The convolution operation involves filter *w* ∈ *R*^*hk*^, which will be applied to a window containing *h* word length to generate new features. For example, the feature *c*_*i*_ is extracted from a sequence with a window length of *X*_*i*:*i*+*h*−1_. The calculation process is as follows:

$$\begin{array}{c} c_{i}=f\left(w_{i}\cdot X_{i:i+h-1}+b_{i}\right) \end{array}$$ (5)

Where *b*_*i*_ ∈ *R* is a bias term, and function *f* represents a nonlinear function, such as a hyperbolic tangent function. Apply this filter to the possible word window [*X*_1:*h*_, *X*_2:*h*+1_, …, *X*_*n*−*h*+1:*n*_] to generate a feature map:

$$\begin{array}{c} c=[c_{1},c_{2},\ldots ,c_{n-h+1}],\;\;\;\;\;c\in R^{n-h+1} \end{array}$$ (6)

In order to capture the most important features from each feature map, this paper uses maximum pooling on the feature map. The calculation process is as follows:

$$\begin{array}{c} s^{*}=Max\left(c\right) \end{array}$$ (7)

Some research work has shown that the intermediate context contains the most relevant semantic information for relation classification, and the intermediate context can also be combined with the left and right contexts as the input of the model [43–45]. Convolution and maximum pooling are used to extract semantic information related to relation classification from the middle context and the left and right contexts respectively. The sequence *S* is divided into five parts by the target entity pair, namely left context [*H*_1_, *H*_2_, …, *X*_*i*−1_], *Entity*_1_ [*H*_*i*_, *H*_*i*+1_, …, *X*_*j*_], middle context [*H*_*j*+1_, *H*_*j*+2_, …, *X*_*p*−1_], *Entity*_2_ [*H*_*p*_, *H*_*p*+1_, …, *X*_*q*_] and right context [*H*_*q*+1_, *H*_*q*+2_, …, *X*_*n*_]. In order to extract useful semantic information for classification tasks from the middle context and the left and right contexts, this paper performs convolution and maximum pooling operations on [*H*_1_, *H*_2_, …, *X*_*i*−1_], [*H*_*j*+1_, *H*_*j*+2_, …, *X*_*p*−1_] and [*H*_*q*+1_, *H*_*q*+2_, …, *X*_*n*_], respectively. The calculation formula process is as follows:

$$\begin{array}{c} S_{1}^{*}=Max\left(Convolution\left(\left[H_{1},H_{2},\ldots ,X_{i-1}\right]\right)\right) \end{array}$$ (8)

$$\begin{array}{c} S_{2}^{*}=Max\left(Convolution\left(\left[H_{j+1},H_{j+2},\ldots ,X_{p-1}\right]\right)\right) \end{array}$$ (9)

$$\begin{array}{c} S_{3}^{*}=Max\left(Convolution\left(\left[H_{q+1},H_{q+2},\ldots ,X_{n}\right]\right)\right) \end{array}$$ (10)

Among them, $S_{1}^{*}$, $S_{2}^{*}$ and $S_{3}^{*}$ indicate the output after convolution and maximum pooling, Convolution indicates one-dimensional convolution, and Max indicates maximum pooling. Convolution and pooling are used to extract features of context subsequences, even if it contains few words, such as one word.

### The objective function

After the above six feature vectors $H_{0}^{*}$, $H_{1}^{*}$, $H_{2}^{*}$, $S_{1}^{*}$, $S_{2}^{*}$ and $S_{3}^{*}$, are spliced, a fully connected layer is added to obtain the relation vector representation *r*. The calculation process is as follows:

$$\begin{array}{c} r=W_{r}\left(concat\left(H_{0}^{*},S_{1}^{*},H_{1}^{*},S_{2}^{*},H_{2}^{*},S_{3}^{*}\right)\right)+b_{r} \end{array}$$ (11)

Among them, *W*_r_ ∈ *R*^|*Y*|×6*d*^ represents the number of relation categories.

For a given sequence *S*, after the relation representation vector *r* is obtained in this paper, the probability distribution ${\tilde{y}}^{\left(i\right)}$ of the relation prediction is obtained through the SoftMax layer. The standard SoftMax function definition is shown in Eq (12), and the calculation formula of probability distribution is shown in Eq (13):

$$\begin{array}{c} \sigma {\left(X\right)}_{i}=\frac{e^{x_{i}}}{\sum_{j=1}^{K}e^{x_{i}}}\;\;\;\;for\;\;i=1,\ldots ,K\;and\;X=\left(x_{1},\ldots ,x_{K}\right)\in R^{K} \end{array}$$ (12)

$$\begin{array}{c} {\tilde{y}}^{\left(i\right)}=\sigma \left(W_{i}*r^{\left(i\right)}+b_{i}\right) \end{array}$$ (13)

Where ${\tilde{y}}^{\left(i\right)}\in Y$ is the relation type of the target, *W*_*r*_ ∈ *R*^|*Y*|×*d*^, *b*_*r*_ ∈ *R*^|*Y*|^.

The cross-entropy loss function is:

$$\begin{array}{c} J\left(Y|\left(X,\theta \right)\right)=-\frac{1}{\left|Y\right|}\sum_{i=1}^{\left|Y\right|}[y_{i}*\log\left({\tilde{y}}^{\left(i\right)}\right)]+w_{\theta }{∥\theta ∥}^{2} \end{array}$$ (14)

Where *X* represents the input sequence vector, and *Y* represents the one-hot encoding relation category vector.

### The focal loss function

Considering that there is still category imbalance in relation extraction tasks, this paper optimizes the model through imbalanced learning methods.

In 2017, a new loss function [17] is proposed to solve one-stage object detection, and there was a great imbalance between foreground and background categories during training (for example, 1:100). The effect of this method is very obvious and exceeds the accuracy of the most advanced secondary detectors available. This method uses a special loss function, i.e., Focal Loss, to control the misclassification cost between samples. The loss function is a focal loss function with balanced variables:

$$\begin{array}{c} FL\left(p_{t}\right)=-\alpha_{t}{\left(1-p_{t}\right)}^{\gamma }\log\left(p_{t}\right) \end{array}$$ (15)

Among them, *α*_*t*_ ∈ [0, 1] is called the weighting factor, *γ* is called the focusing parameter, $y\in \left[-1,1\right],\;p_{t}{=\{}_{1-p\;\;\;\;otherwise}^{p\;\;\;\;\;\;if\;y=1}$ and *p* are the predicted probability of label *y* = 1 by the model.

The research objects in this paper have unbalanced categories, so the cross-entropy loss function needs to be modified to adapt to the task of relation extraction. This paper proposes the focal loss function with *L*2 regular term as follows Eq (16):

$$\begin{array}{c} \begin{array}{c} J\left(\theta \right)=-\frac{1}{m}\sum_{i=1}^{m}[y^{\left(i\right)}\alpha {\left(1-{\tilde{y}}^{\left(i\right)}\right)}^{\gamma }\log\left({\tilde{y}}^{\left(i\right)}\right)+\left(1-y^{\left(i\right)}\right)\left(1-\alpha \right){\left({\tilde{y}}^{\left(i\right)}\right)}^{\gamma }\log\left(1-{\tilde{y}}^{\left(i\right)}\right))]\\ +w_{\theta }{∥\theta ∥}^{2} \end{array} \end{array}$$ (16)

Among them, *θ* represents all the parameters in the model, and the weighting vector *α* is a multi-category proportional weight vector, giving a new ratio of positive and negative samples to achieve a balance. For example, when *α* is 0.25, that is, the proportion of positive samples is smaller than that of negative samples. This is because there are more negative samples and easy to divide. Although only adding *α* can balance the importance of positive and negative samples, it cannot solve the problem of simple and difficult samples. Therefore, another parameter *γ*(*γ* > 0) needs to be introduced. The purpose is to reduce the loss of easy-to-classify samples, so that the model can focus more on difficult-to-classify samples during training. For example, *γ* is 2, for positive samples, the sample whose model prediction result is 0.95 must be a simple and easy to divide sample, so the *γ* power of (1-0.95) will be small, and the loss function value will become smaller. Similarly, when the predicted probability of the model is 0.3, the loss function value of the sample is relatively larger. For negative samples, the loss function value of the sample with a model prediction result of 0.1 should be much smaller than the sample loss function with a prediction result of 0.7. When the predicted probability is 0.5, the loss is only reduced by 0.25 times, so the model pays more attention to this indistinguishable sample. By reducing the impact of the loss function value of simple samples, the effect of predicting the probability of difficult samples is more effective.

## The results and analysis

### The datasets and environment

The SemEval-2010 task-8 contains 10717 annotated data, 8000 of which are used as training set and 2717 as test set. There are nine direct relation types and one artificial other relation type in the dataset. Among the relations, nine direct relation types are Cause-Effect, Component-Whole, Entity-Destination, Product-Producer, Entity-Origin, Member-Collection, Message-Topic, Content-Container and Instrument-Agency. The dataset also considers the direction, for example, Component-Whole (*e*1, *e*2) and Whole- Component (*e*2, *e*1) belong to different relation types, so 19 relation types are considered in this experiment.

The SemEval-2018 task-8 is the first database to annotate malware reports, and uses MAEC to annotate APT reports. The MAEC framework is a structured language to describe the attributes and characteristics of malware. At present, the data set is composed of 84 APT reports, which has four relation types, ActionObj, SubjAction, ModObj and ActionMod. This paper focuses on the task of relation extraction at the sentence level. The report is segmented to sectences, so that each sentence contains only one pair of entities and corresponding relation categories. Finally, 10182 pieces of data are obtained, containing 8919 pieces training data and 1263 pieces testing data. The training data has 2867 pieces of Actionobj, 2323 pieces of SubjAction, 1897 pieces of ModObj and 1832 pieces of ActionMod. The testing data has 420 pieces of Actionobj, 309 pieces of SubjAction, 264 pieces of ModObj and 270 pieces of ActionMod.

According to the official evaluation index of SemEval-2010 Task 8, this paper uses the macro-average F1 value (Macro-F1) to evaluate the model for the nine direct relation types. The calculation formula of the macro average F1 value is as follows:

$$\begin{array}{c} MacroF1=\frac{1}{n}\sum_{i=1}^{n}F1_{i} \end{array}$$ (17)

Where *n* is the total number of relation categories.

Related algorithms in this paper all need to involve the BERT model, and the experimental environment is shown in Table 4. For the pre-trained BERT model, select the official Bert-large-uncased version to train. The detail of Bert has 24-layers, 1024-hidden, 16-heads and 340M parameters.

**Table 4:** The experimental environment and illustration.

Lab Environment	Description
Operating system	Ubuntu 16.04
GPU	RTX 2080Ti
Video memory	11G
Hard disk	1T
Programming language	Python3.6
Deep learning framework	PyTorch1.1
Bert version	Bert-large-uncased

### The analysis of results

In order to verify the effect of piecewise convolution and focal loss function on relation extraction, this paper sets up multiple sets of control experiments on the SemEval-2010 Task 8 dataset and SemEval2018 Task8 dataset, to illustrate the impact of the two modules on the final relation extraction results.

- **SemEval-2010 Task 8 Dataset**.

In Experiment I, this paper directly uses the last layer of the BERT model hidden layer output, that is, the vector representation corresponding to the identifier [*CLS*] at the beginning of the sentence, and then passes through a fully connected layer to obtain the relation category probability distribution. This experiment uses the cross-entropy function (Cross Entropy) as the loss function.

Unlike Experiment I, the focal loss function was used as the loss function in Experiment II.

In Experiment III, this paper obtains the coded representation [*H*_*i*_, *H*_*i*+1_, …, *X*_*j*_] of *Entity*_1_ in the sentence according to the identifier $ in the sentence, and obtains the coded representation [*H*_*p*_, *H*_*p*+1_, …, *X*_*q*_] of *Entity*_2_ in the sentence according to the identifier # in the sentence. On the basis of experiment, information of *Entity*_1_ and *Entity*_2_ is added.

Unlike Experiment III, the focal loss function was used as the loss function in Experiment IV.

In experiment V, this paper uses convolutional pooling operation to obtain feature information useful for classification results from noise segment $\left[S_{1}^{*},S_{2}^{*},S_{3}^{*}\right]$. On the basis of Experiment III, the above-mentioned characteristic information was added to Experiment V.

In Experiment VI, unlike Experiment V, the focal loss function is used as the loss function.

The core parameter selection involved in the control experiment and the parameter selection of the training process are shown in Table 5. It has been proved that the natural language pre-training model can learn the general language effectively, indicating that Bert, as the most outstanding representative, has achieved good results in various NLP tasks. So, we use the pre-trained Bert model to extract the semantic information from the sentence. And the longest accepted length of sequence for Bert-base model is 512, the text sequence can have one or two clauses separated by [SEP] and the sequence begins with [CLS]. Focus on the training dataset, we train the pre-trained model by fine tune. The hyper-parameters setting process are as follows: In this experiment part, the auto-optimized method is used to optimize the hyper-parameters. First, the Max sentence length after Tokenization, Batch size for training, Number of training epochs and Dropout rate are fixed in this dataset. The remaining hyper-parameters are selected by auto-optimized method. During the training, the parameters are auto search in the section, the upper and lower limits of the section are set empirically. The section of Initial learning rate for Adam is [10^−1^, 10^−5^], the section of *L*2 regularization coefficient is also [10^−1^, 10^−5^], the section of window size is [2, 5], the section of Gamma is [0, 6], the Alpha’s section is set as proportion of all types of relations, and fine tuning based on the proportion. During the training processing, the random search optimizer is adopted to select the hyper-parameters from the spaces. Because of the spaces are neither continuous nor differentiable, the random search algorithm first randomly selects the hyper-parameters to be evaluated and repeat the process until the performance are satisfied. After training, the hyper-parameters are defined.

**Table 5:** The hyper-parameters of Bert for fine tune in SemEval-2010 Task-8 dataset.

Parameters	Values
Max sentence length after Tokenization	128
Batch size for training	8
Initial learning rate for adam	2E-5
Number of training epochs	5
L2 regularization coefficient	5E-3
Dropout rate	0.1
Window_size	2
Gamma	2
Alpha	[1, 4, 2, 14, 3, 4, 4, 4, 9, 3, 9, 1, 100, 3, 3, 14, 2, 3, 9]

#### Piecewise convolution optimization

**Fig 3:**
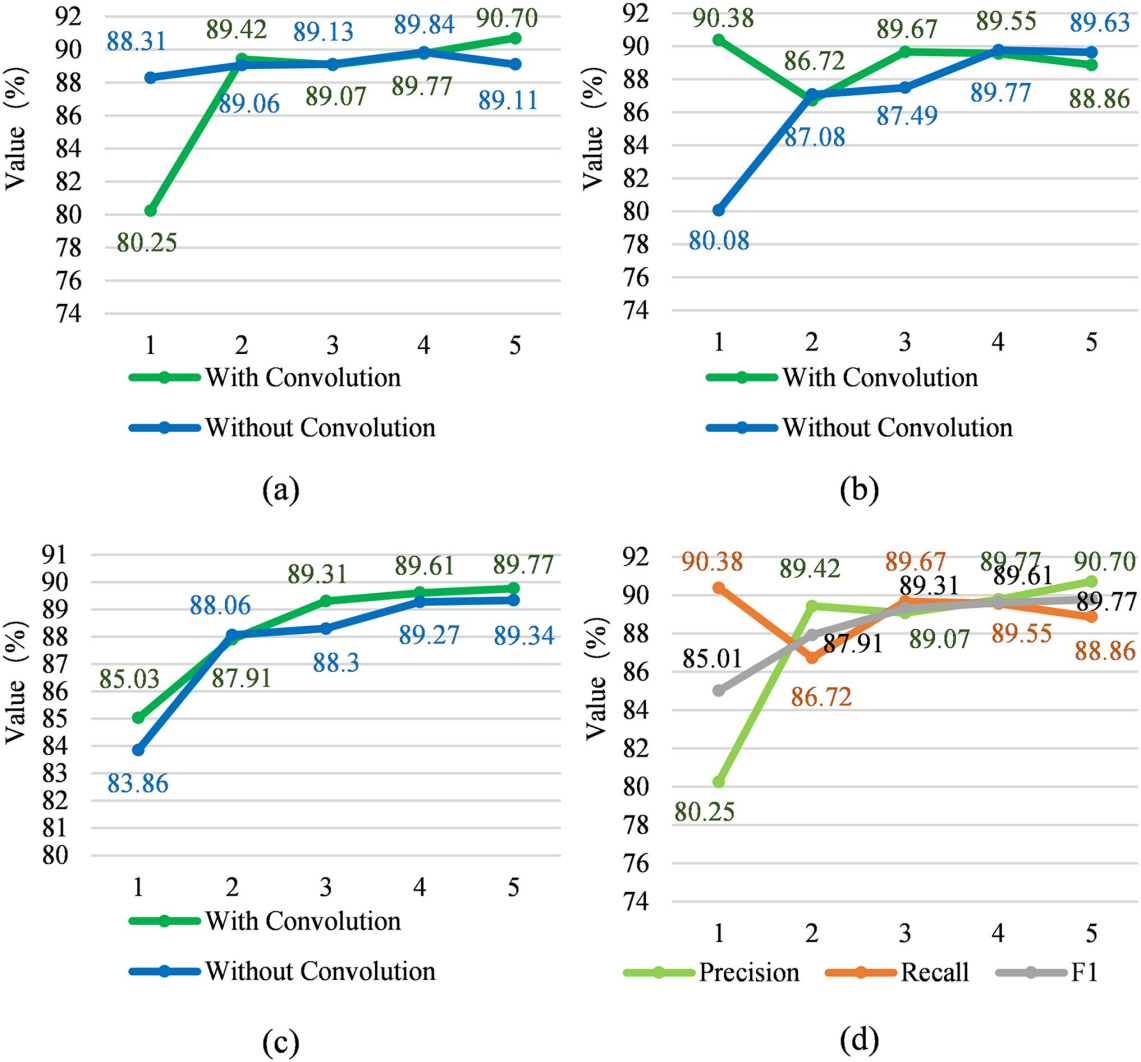
Precision, recall and F1 rate of piecewise convolution and pooling module.

Fig 3 shows the comparison of the three reference values of Precision, Recall, and F1 with and without the piecewise convolution on the training set, and the piecewise convolution with the training batch. The trend of change is shown in Fig 3. The Fig 3(a) shows the precision rate change of adding piecewise convolution and not adding piecewise convolution, and Fig 3(b) shows the recall rate of adding piecewise convolution and not adding piecewise convolution. Fig 3(c) shows the F1 change with and without piecewise convolution. Fig 3(d) shows the precision rate, recall rate and F1 change with piecewise convolution. The horizontal axis is the number of iterations (epoch). According to the comparison between Fig 3(a) and 3(b), comparing the precision rate or recall rate separately, it is found that the effect of piecewise convolution is not obvious, because the curve changes greatly. However, according to the F1 value curve in Fig 3(c), the curve with piecewise convolution is basically the same as the curve without piecewise convolution. It can be said that the effect is more obvious. Compare with the model without piecewise convolution, the model with piecewise convolution is 9.13% lower, 0.4% higher, 0.07% lower, 0.08% lower and 1.78% higher than without in accuracy from experiment I to V; and 12.86% higher, 0.41% lower, 2.49% higher, 0.25% lower and 0.86% lower than without in recall from experiment I to V; and 1.4% higher, 0.17% lower, 1.14% higher, 0.38% higher and 0.48% higher than without in F1 from experiment I to V.

Compare with the model without piecewise convolution, the model with piecewise convolution is 9.13% lower, 0.4% higher, 0.07% lower, 0.08% lower and 1.78% higher than without in accuracy from experiment I to V; and 12.86% higher, 0.41% lower, 2.49% higher, 0.25% lower and 0.86% lower than without in recall from experiment I to V; and 1.4% higher, 0.17% lower, 1.14% higher, 0.38% higher and 0.48% higher than without in F1 from experiment I to V. In addition, according to the precision rate, recall rate and F1 value of each batch in Fig 3(d), the stability of piecewise convolution is not strong, and the fluctuation range of the curve is very large. The purpose of piecewise convolution is to extract useful semantic information from the noise segment, which means that the result is affected by the intensity of the noise in the sequence.

#### Optimization of focal loss function

**Fig 4:**
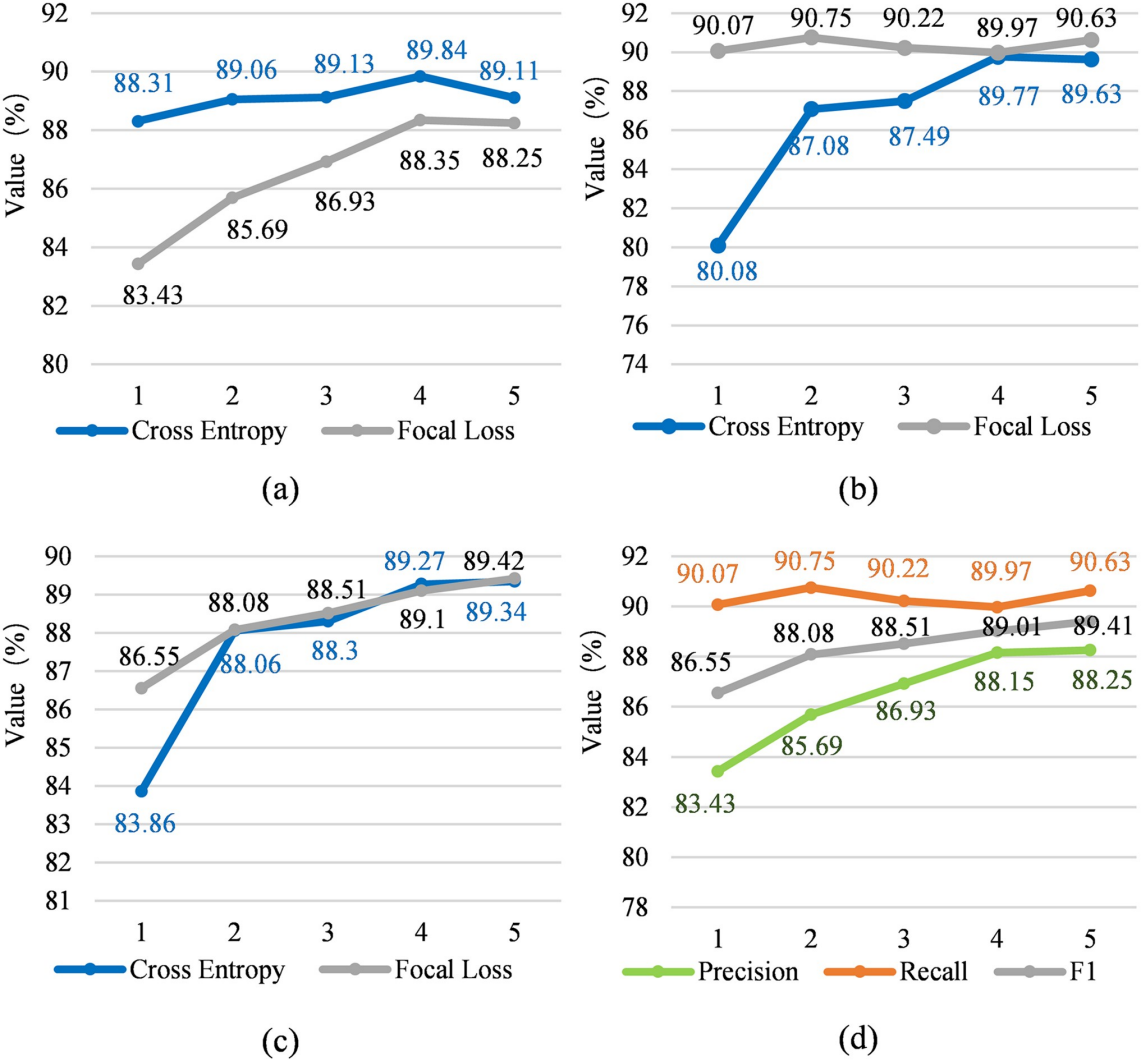
Precision, recall and F1 rate of focal loss module.

Fig 4 shows the comparison of the focus loss function structure and the three reference values of Precision, Recall, and F1 of the cross-entropy loss function on the training set, and the trend of the focus loss function with the training batch. Fig 4(a) shows the change in accuracy of the two loss functions of focus loss and cross entropy loss, Fig 4(b) shows the change in recall rate of the two loss functions of focus loss and cross entropy loss, Fig 4(c) shows the F1 change of the two loss functions of focus loss and cross entropy loss. Fig 4(d) shows the precision rate, recall rate and F1 change of the focus loss function. The horizontal axis is the number of iterations (epoch). Compare with the model without the focal loss, the model with the focal loss is 5.53% lower, 3.78% lower, 2.47% lower, 1.66% lower and 0.97% lower than without in accuracy from experiment I to V; and 12.48% higher, 4.21% higher, 3.12% higher, 0.22% higher and 1.12% higher than without in recall from experiment I to V; and 3.2% higher, 0.02% higher, 0.24% higher, 0.19% lower and 0.09% higher than without in F1 from experiment I to V. According to the comparison between Fig 4(a) and 4(b), the focus loss function mainly improves the recall rate. And it is found that changes in the magnitude of the loss of function of the focus is very smooth, indicating that the performance of the function is relatively stable.

#### Analysis of control experiment results

The final results of the six groups are shown in Table 6. The metric of F1 is used to measure the accuracy of classification model in statistics. It considers the accuracy and recall of the classification model at the same time and can be regarded as a harmonic average of the accuracy and recall of the model. Therefore, the F1 metric is used as the main evaluation index of the following experiments.

**Table 6:** The comparison on test results of different modules.

Number	Method	Loss Function	F1
I	BERT-based without Entity Information	Cross Entropy	87.99
II	BERT-based without Entity Information	Focal Loss	89.32
III	BERT with Entity Information	Cross Entropy	89.25
IV	BERT with Entity Information	Focal Loss	89.42
V	BERT with Entity, convolution and max-pooling	Cross Entropy	89.77
VI	BERT with Entity, convolution and max-pooling (our model)	Focal Loss	**89.95**

From the Table 6, three pairs of controlled experiments are made, the difference of first pair (Experiment I and Experiment II) is the loss function, the second pair and third pair have the same difference. From the three pairs experiments, the modified focal loss function can enhance the performance significantly, it illustrates that the improvement is effect. From the Experiment II, IV and VI, the differences are entity information and piecewise convolution. From these experiments, the results illustrate that the remaining segments of corpus contains relation information. Then hidden information can greatly assist the relation extraction. In addition, according to the comparison between Experiment V and Experiment III, Experiment VI and Experiment IV, the two sets of control experiments show that the piecewise convolution operation improves the experimental results by 0.52% and 0.19%, which proves that the piecewise convolution operation proposed in this paper is effective for the relation extraction model. The proposed model (Experiment VI) compares with the original BERT (Experiment I), the performance improved 2.3% on F1.

**Fig 5:**
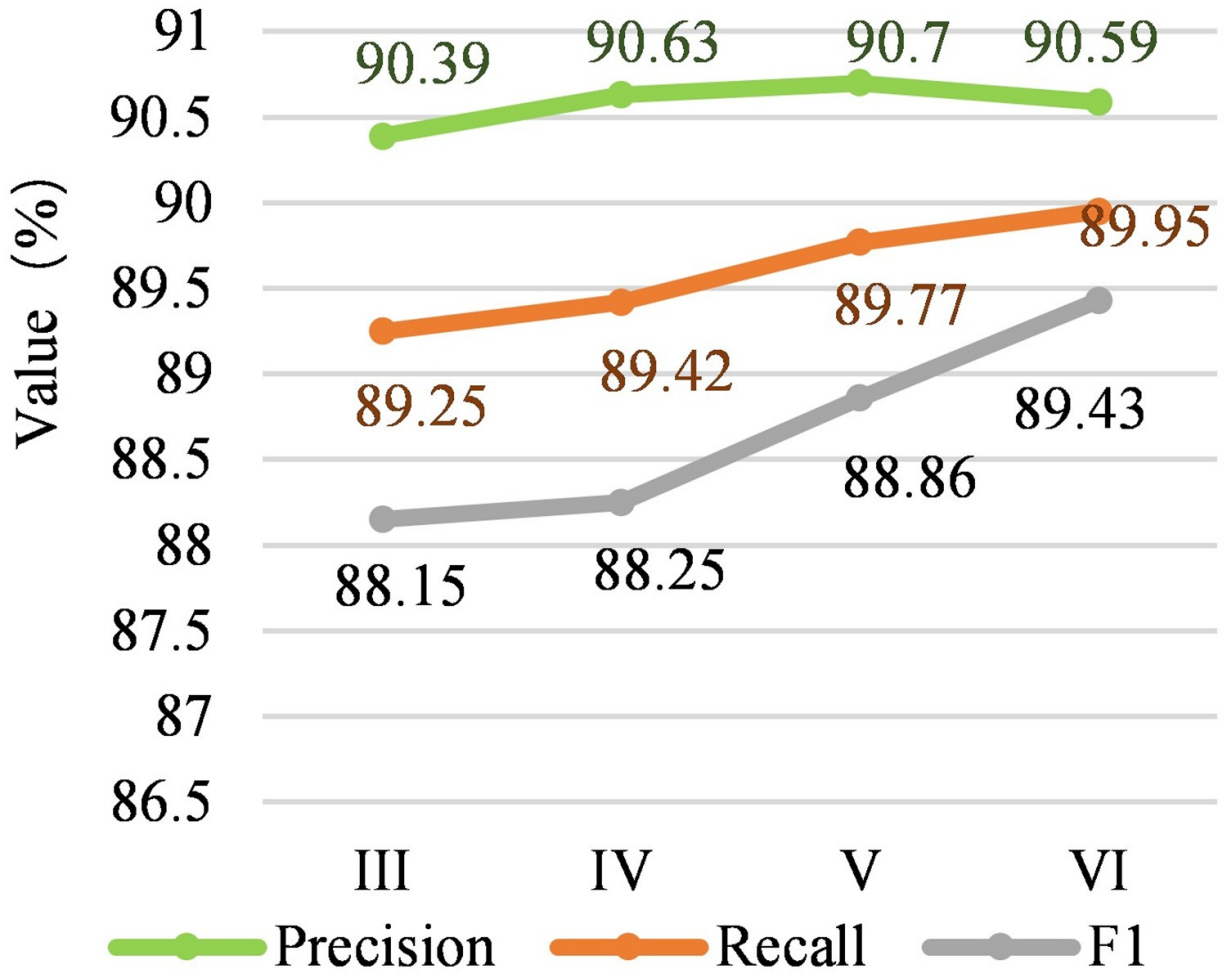
Comparison on precision, recall, F1 results of different modules.

The comparative experiments are done from the three perspectives of precision rate, recall rate and F1 value. The experimental results are shown in Fig 5. The horizontal axis (III, IV, V, VI) is the model represented by the number in Table 7, and the vertical axis represents the value. The proposed model is 2.5% higher than R-Bert in precision, 1.66% higher in recall and 2.04% higher in F1. It can be seen from Fig 5 that the overall trend of the three curves shows an upward trend, indicating that Experiments IV, V, and VI are generally better than Experiment III. The Experiment VI is 0.22% higher, 0.04% lower and 0.12% lower in precision than Experiment III to V, and 0.78% higher, 0.59% higher and 0.2% higher in recall, and 1.45% higher, 1.34% higher and 0.64% higher in F1. At the same time, for the analysis of measurement indicators, the fluctuation range of F1 curve is more stable than the two curves of precision rate and recall rate, indicating that the measurement indicator F1 value is more suitable for measuring the model in this paper.

**Table 7:** Comparison of R-BERT and proposed model.

Relationship category	Precision Rate		Recall Rate		F1 Value	
	R-BERT	Ours	R-BERT	Ours	R-BERT	Ours
Cause-Effect	90.91	**93.33**	**94.51**	93.91	92.68	**93.62**
Component-Whole	83.86	**87.23**	84.94	**89.74**	84.39	**88.47**
Content-Container	**89.8**	88.24	91.67	**93.75**	90.72	**90.91**
Entity-Destination	91.69	**93.86**	**94.52**	94.18	93.09	**94.02**
Entity-Origin	88.1	**92.43**	86.05	**89.92**	87.06	**91.16**
Instrument-Agency	82	**86.43**	**78.85**	77.56	80.39	**81.76**
Member-Collection	84.36	**88.33**	87.98	**90.99**	86.13	**89.64**
Message-Topic	**89.13**	87.46	**94.25**	93.49	**91.62**	90.37
Product-Producer	85.48	**87.61**	89.18	**91.77**	87.29	**89.64**
Average	87.26	**89.44**	89.11	**90.59**	88.15	**89.95**

**Fig 6:**
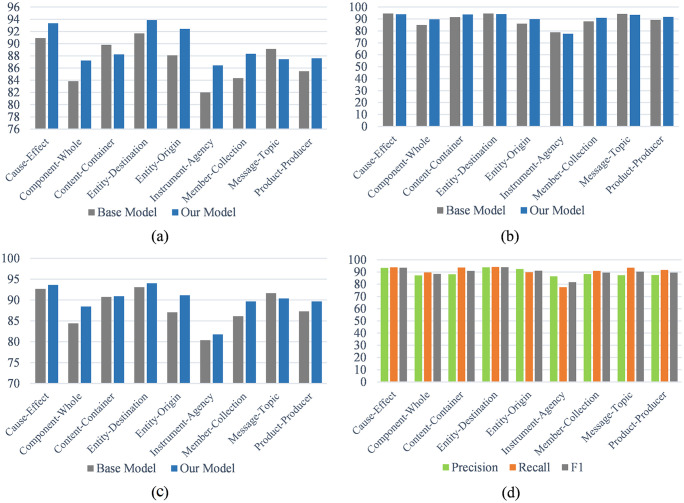
The influence of this module on the precision, recall and F1 of every class.

We calculated the precision rate, recall rate and F1 value of each classification category. The R-BERT [1] model was selected as the baseline model (base model) control. The results are shown in Fig 6 and Table 7. In Fig 6, the Fig 6(a) shows the comparison of the precision rate of each category of the baseline model and the model of this paper. Fig 6(b) shows the comparison of the F1 value of each category of the baseline model and this model, Fig 6(c) shows the comparison of the recall rate of each category of the baseline model and the model of this paper. Fig 6(d) shows the comparison of the precision rate, recall rate and F1 value of each category of the model of this paper. According to Fig 6(a) and 6(b), it can be seen that the model in this paper has a greater improvement in accuracy and F1 value. Regarding the F1 value, in addition to one category, other categories have improved, especially the F1 value of the Component-Whole, Entity-Origin, Member-Collection, and Product-Producer categories increased by 4.12%, 4.1%, 3.51% and 2.35% respectively. According to the comparison of Fig 6(a), 6(b) and 6(c), the model in this paper greatly improves the precision rate of the relation category, however, the improvement of the recall rate is not very obvious.

- **SemEval-2018 Task 8 Dataset**.

In this paper, 4 sets of control experiments are set up on the SemEval-2018 Task-8 data set. Since the data volume of this data set is larger than that of SemEval-2010 Task-8 and the number of relation categories is small, the effect on this data set is better. The experiment number is explained as follows:

1. In experiment I, the BERT model is used and the feature information of *Entity*_1_ and *Entity*_2_ is added, and the cross-entropy loss function is used.
2. In Experiment II, unlike in Experiment I, the focal loss function is used as the loss function.
3. In Experiment III, the convolutional pooling operation is used to obtain feature information useful for the classification result from the noise segment. On the basis of Experiment I, the above-mentioned feature information was added to Experiment V.
4. In Experiment IV, unlike in Experiment III, the focal loss function is used as the loss function.

The number of experimental hyperparameters and the selection of the core parameters of the model training process are shown in Table 8. The hyper-parameters of the SemEval-2018 Task-8 dataset is fine tuning based on Table 5.

**Table 8:** The hyper-parameters of Bert for fine tune in SemEval-2018 Task-8 dataset.

Parameters	Value
Max sentence length after Tokenization	128
Batch size for training	8
Initial learning rate for Adam	2E-05
Number of training epochs	3
*L*2 regularization coefficient	5E-03
Dropout rate	0.1
Window size	2
Gamma	2
Alpha	[2, 3, 2, 3]

**Fig 7:**
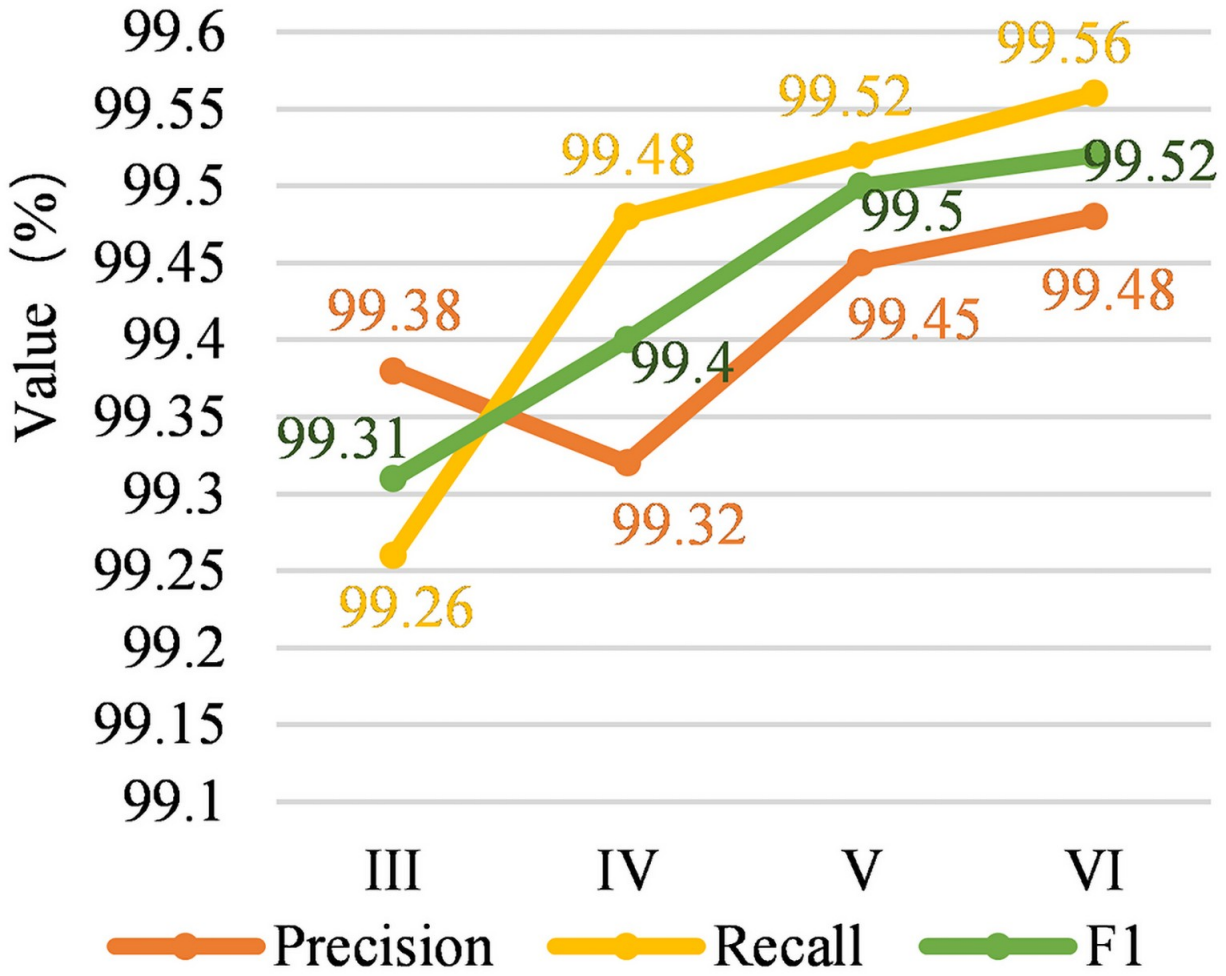
Comparison on precision, recall, F1 results of different modules.

The experimental results are shown in Fig 7. In Fig 7, the three curves from top to bottom are recall rate, F1 value and precision rate. The horizontal axis (I, II, III, IV) is the experiment number represented above, and the vertical axis represents the value. The changes of the three curves are basically consistent with Fig 5. The recall rate curve is the highest, followed by the F1 value curve, and finally the precision rate curve. It can be seen from Fig 7 that the values of the three curves are very close and the values are very large, which also shows that the number of samples has a direct impact on the experimental results. We found that the relation category distribution of the SemEval-2018 Task-8 data set is general balanced, which also shows that the three curves basically change within the range of 99.26%—99.56%. So, the impact of the focal loss function is not obvious in this dataset.

**Fig 8:**
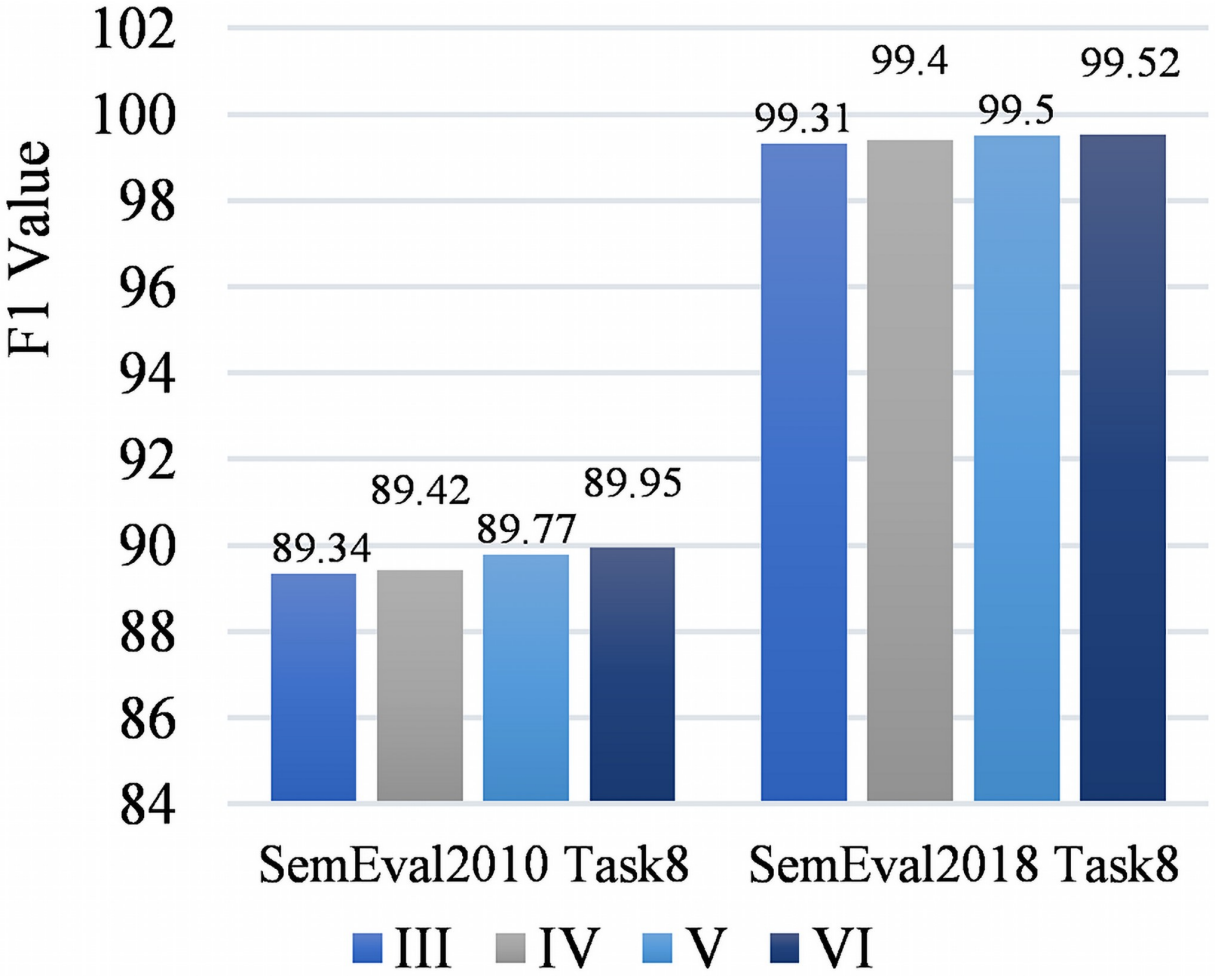
F1 variation diagram of different model on two datasets.

Fig 8 shows the F1 value changes of the four models on the two data sets. From Fig 6 we can find that there are certain differences in the experimental results on different data sets. The model in this paper has obtained the highest F1 values on the SemEval-2010 Task-8 and SemEval-2018 Task-8 data sets, which are 89.95% and 99.52%. The F1 value of this model on the SemEval-2010 Task-8 and SemEval-2018 Task-8 datasets have improved compared with the baseline model F1 values by 0.61% and 0.21%. The F1 value of the piecewise convolution operation proposed in this paper is increased on the SemEval-2018 Task-8 and SemEval-2010 Task-8 datasets by 0.1% and 0.35% respectively; the focal loss function proposed in this paper operates on SemEval-2018 Task-8 and SemEval-2010 Task-8. The F1 value on the Task-8 data set has been increased by 0.09% and 0.18% respectively.

- **Comparison with Existing Methods**.

The relation extraction model proposed in this paper is compared with the current multiple relation extraction models released based on the SemEval-2010 Task-8 data set, including RNN, CNN, FCM, CR-CNN, Attention-CNN, Multi-Attention-CNN, Hierarchical Attention Bi-LSTM, Entity Attention Bi-LSTM, R-BERT and Matching-the-Blanks BERT. Table 9 shows the comparison results between this paper and the existing methods. The MACRO F1 value of the model proposed in this paper is 89.95%, which exceeds the existing methods.

**Table 9:** The comparison of results of different models.

Methods	F1 Value
RNN (proposed in 2012)	77.6
CNN (proposed in 2014)	82.7
FCM (proposed in 2014)	83
CR-CNN (proposed in 2015)	84.1
Attention CNN (proposed in 2016)	84.3
Multi-Attention-CNN (proposed in 2016)	88
Hierarchical Attention Bi-LSTM (proposed in 2016)	84.3
Attention Bi-LSTM (proposed in 2016)	84
Entity Attention Bi-LSTM (proposed in 2019)	85.2
R-BERT (proposed in 2019)	89.25
Matching-the-Blanks BERT (proposed in 2019)	89.5
Our model	**89.95**

Through the Tables 6 and 9 results, the proposed model is superior than the exiting methods. It illustrates that the remaining segments of corpus between the entities contain the valuable hidden information for relation extraction. And the effect of piecewise convolution and modified focal loss function are verified in the experiments.

1. In the Table 6, compare with the Experiment I and III, II and IV, the entities and semantic information can enhance the relation extraction performance, which illustrate that the relation information not only exist in entities but also in semantic information between the entities.
2. Compare the Experiment III and IV, V and VI in Table 6, from the results, the piecewise convolution can help extract the features from the entities and semantic information in sentence, which can enhance the extraction accuracy. The piecewise convolution has fewer parameters than traditional convolution while ensuring the performance, so, the network can be designed more deeper when uses the piecewise convolution.
3. From the three pairs experiments, Experiment I and II, III and IV, V and VI, these experiments compare the loss function in the model. From the F1 metric, the focal loss function can alleviate the problem of sample imbalance in the dataset. By assigning more weight to the relationship category with less samples, it can better learn the features of the category and improve the extraction accuracy of each category.

The methods listed in Table 9 are based CNN, LSTM and BERT. Compare with the existing method, how does the added piecewise convolution affect the execution efficiency of the proposed model. The time complexity analysis of the proposed model is given. The proposed relation extraction model is a sequential model, the sentences are encoded by BERT firstly, and then the piecewise convolution extracts the features from the remaining segments of corpus. So, the time complexity of proposed model is additive by the BERT and the piecewise convolution.

The total time complexity of the proposed relation extraction model can be represented as Eq (18):

$$\begin{array}{c} Time∼O\left(n^{2}\cdot d\right)+O\left(M\cdot K\right)∼O\left(n^{2}\cdot d\right) \end{array}$$ (18)

In Eq (18), *O*(*n*^2^ ⋅ *d*) is the time complexity of BERT, and *O*(*M* ⋅ *K*) is the time complexity of piecewise convolution. The time complexity of BERT is mainly concentrated in the Self-Attention module, and the complexity of the Self-Attention can represent the BERT. The *n* is the sequence length of the input, and the *d* is the dimension of embedding. Among the Eq (18), the *M* is the sequence length of the feature map (encoding vector) after convolution; and the kernel size *K* which used in the proposed model called windows size (because the input of convolution layer is one-dimension data, so the kernel called window).

So, compared with the existing models, the time complexity of the proposed model does not increase significantly.

## Conclusion

As for an important sub-task in information extraction, relation extraction is the basis of many subsequent tasks. However, the relation extraction models of convolutional neural network and recurrent neural network do not make good use of the semantic information of the sequence, and even adopt the artificially features for feature extraction. Meanwhile, the semantic information of entities and generalization ability of model are ignored when extracting the relation. Based on the existing work of relation extraction, the Bert model which proposed by Google can able to learn the word dependencies within sequences well, so, the pre-trained Bert model in this paper is used to vector the features of entities and semantic information between entities. Then obtain the entity representation after Bert vectorization, the residual noise corpus is segmented and convoluted to extract useful semantic information, and finally the entity and relationship are classified. As for generalization ability, the focal loss is used to optimized the model by assigning different weights in training. The proposed model is compared with several methods recently published for Semeval-2010 Task 8 dataset, and the metric of F1 can reach 89.95%.

In addition, the research of semantic relation extraction needs to be further improved, such as improving the extraction precision, the neural network architecture and using the distant supervision. Distant supervision can provide labels for data from external knowledge base, and save the trouble of manual labeling, so we would use the distant supervision for relation extraction in our future work.
